# A Lesion-Proof Brain? Multidimensional Sensorimotor, Cognitive, and Socio-Affective Preservation Despite Extensive Damage in a Stroke Patient

**DOI:** 10.3389/fnagi.2016.00335

**Published:** 2017-01-10

**Authors:** Adolfo M. García, Lucas Sedeño, Eduar Herrera Murcia, Blas Couto, Agustín Ibáñez

**Affiliations:** ^1^Laboratory of Experimental Psychology and Neuroscience, Institute of Cognitive and Translational Neuroscience, INECO Foundation, Favaloro UniversityBuenos Aires, Argentina; ^2^National Scientific and Technical Research Council (CONICET)Buenos Aires, Argentina; ^3^Faculty of Elementary and Special Education, National University of CuyoMendoza, Argentina; ^4^Departamento de Estudios Psicológicos, Universidad IcesiCali, Colombia; ^5^Universidad Autónoma del CaribeBarranquilla, Colombia; ^6^Center for Social and Cognitive Neuroscience, School of Psychology, Universidad Adolfo IbáñezSantiago de Chile, Chile; ^7^Centre of Excellence in Cognition and its Disorders, Australian Research CouncilSydney, NSW, Australia

**Keywords:** stroke, bilateral lesions, multimodal preservation, cognitive reserve

## Abstract

In this study, we report an unusual case of mutidimensional sensorimotor, cognitive, and socio-affective preservation in an adult with extensive, acquired bilateral brain damage. At age 43, patient CG sustained a cerebral hemorrhage and a few months later, she suffered a second (ischemic) stroke. As a result, she exhibited extensive damage of the right hemisphere (including frontal, temporal, parietal, and occipital regions), left Sylvian and striatal areas, bilateral portions of the insula and the amygdala, and the splenium. However, against all probability, she was unimpaired across a host of cognitive domains, including executive functions, attention, memory, language, sensory perception (e.g., taste recognition and intensity discrimination), emotional processing (e.g., experiencing of positive and negative emotions), and social cognition skills (prosody recognition, theory of mind, facial emotion recognition, and emotional evaluation). Her functional integrity was further confirmed through neurological examination and contextualized observation of her performance in real-life tasks. In sum, CG's case resists straightforward classifications, as the extent and distribution of her lesions would typically produce pervasive, multidimensional deficits. We discuss the rarity of this patient against the backdrop of other reports of atypical cognitive preservation, expound the limitations of several potential accounts, and highlight the challenges that the case poses for current theories of brain organization and resilience.

## Introduction

The mind-brain association, as conceived in clinical neuroscience and neuropsychology, is an abstract generalization. In working with multi-participant samples, behavioral findings stem from data averages while anatomical results are obtained by transforming brain images into a standard coordinate space. In both cases, strict outlier exclusion criteria are applied, so that atypical patterns are removed from ensuing models. These steps are critical and perhaps unavoidable to characterize the organ's functional organization with some degree of external validity. Indeed, thanks to this approach, replicable associations have been established between damage to circumscribed regions and impairments of specific functions, including motor (Zgaljardic et al., 2003), somatosensory (Meyer et al., 2016), socio-cognitive (Gold and Shadlen, 2007; Ibáñez et al., 2010, 2016b; Couto et al., 2013; Baez et al., 2014, 2016b,c; Melloni et al., 2016), interoceptive (Couto et al., 2015; García-Cordero et al., 2016), executive (Rabinovici et al., 2015; Sedeño et al., 2016), linguistic (Ullman, 2008; Cardona et al., 2014; García and Ibáñez, 2014, 2016; Bocanegra et al., 2015; García, 2015; Melloni et al., 2015; García et al., 2016a,b,c; Abrevaya et al., 2017), and pragmatic (Kaplan et al., 1990; Stemmer, 2008) skills.

However, such well established anatomo-clinical links (and the theoretical views construed around them) are sometimes challenged by unusual individual cases which do not easily fit mainstream models in cognitive neuroscience. Such reports include that of a man who efficiently served as a civil servant although he had progressively lost roughly 75% of his brain (Feuillet et al., 2007), that of a housewife with only mild motor symptomatology despite primary cerebellar agenesis (Yu et al., 2015), or multiple patients exhibiting considerable restitution of language skills following early left hemispheromotomy (e.g., Hertz-Pannier et al., 2002). The same is true of studies showing preserved pre- and post-operative temporal functions in patients with large perisylvian arachnoid cysts (Kunz et al., 1988), although such malformations typically impair various cognitive domains (Wester, 2008). Cases such as these are valuable because, in their exceptionality, they invite us to extend our current conceptions of brain organization, plasticity, and functional compensation, beyond the robust patterns that emerge in typical, averaged, normalized data.

Building on these premises, we present the remarkable case of patient CG, who exhibits widespread sparing of sensorimotor, cognitive, and socio-affective functions despite extensive brain damage acquired in adulthood. In particular, as shown below, CG's unusual pattern of preservation was convergently corroborated through neuropsychological assessment, multiple experimental tasks, neurological examination, and even naturalistic observations of her daily functioning. The case could thus prompt new reflections on the functional organization of various neurocognitive systems.

## Case description

Patient CG is a 44-year-old, right-handed Argentine woman who suffered from adult-onset acquired neurovascular lesions. She reports no individual or familial antecedents of psychiatric or neurological disease, as well as no history of familial sinistrality. She completed 18 years of formal education, is fluent in Spanish and English, and prior to her lesion, she had a managerial position at an international bank.

On September 9, 2011, at age 43, CG experienced intense headaches and nausea, lost consciousness, and was swiftly hospitalized. Computed tomography revealed a subarachnoid hemorrhage (Fisher scale: grade IV; Hunt-Hess scale: grade V) which caused increased intracranial pressure and compromised sulci, cysterns, and fissures bilaterally. Cerebral angiography confirmed a 5-mm dysplastic fusiform aneurysm at the right Sylvian trifurcation, with a smaller incidental aneurysm at the origin of the anterior temporal artery. Upon detection of severe refractory intracranial hypertension, the patient underwent right fronto-parieto-temporal decompressive surgery. Ten days later, CG suffered a cardio-respiratory arrest and regained consciousness after reanimation. She then exhibited severe vasospasm for another ten days leading to extensive damage (delayed cerebral ischaemia) of the right hemisphere which includes frontal, insular, temporo-parietal, and occipital regions as well as striatal areas in the left hemisphere. In addition, a neurological examination revealed a transient, left visual neglect syndrome that lasted 5 days (and which never relapsed in the patient's clinical history). Having spent 41 days in intensive care, she was discharged with a moderate left paresis and the following pharmacological treatment: omeprazole 20 mg qd, aspirin 100 mg qd, enalapril 10 mg bid, and paroxetine 20 mg qd.

Six months later, the patient experienced convulsive status epilepticus, which were treated first with phenytoin (100 mg e/8 h) and then with levetiracetam (1000 mg bid). On December 5, 2012, she underwent craniotomy, with removal of the right frontal, parietal, and temporal bones and implantation of a prothesis and a titanium plaque (pharmacological treatment after surgery included: bisoprolol 7.5 mg qd, enalapril 10 mg bid, quetiapine 12.5 mg qd, and levetiracetam 2000 mg qd). On February 8, 2013, she suffered a second (ischemic) stroke produced by a sudden reswallowing that might have been related to the previous surgery. This resulted in damage to the left anterior insular cortex and its underlying white matter, the putamen, and the dorso-lateral amygdala. Yet, after clinical stabilization, the patient presented neither additional neurological deficits nor cognitive, emotional or behavioral impairments. The only immediate changes she reported were loss of sensitivity on the right hand and a transient form of personality-color synesthesia (Ramachandran et al., 2012; Safran and Sanda, 2015). She was soon discharged to her home, under supervision of a caregiver, and started physical therapy sessions. At this stage, her pharmacological treatment comprised bisoprolol 7.5 mg qd, quetiapine 12.5 mg qd, levetiracetam 1000 mg td and omeprazole 20 mg qd. The patient then agreed to undergo an extensive assessment, including formal neuropsychological evaluations, neurological examinations, and situated observation of her everyday functioning, as reported below.

## Materials and methods

### Participants

The patient and a group of controls completed several tasks tapping domains typically associated with the patient's lesioned areas. The control samples were composed of healthy women with no history of neurological or psychiatric disease. Note that the wide range of tasks we used belonged to various previous (Couto et al., 2013, 2015) and on-going protocols, which did not necessarily involve the same participants. Thus, for each domain assessed, we selected the control group that best matched patient CG in terms of age and education level (for details, see Table 1 through **Table 4**). All participants provided written informed consent in agreement with the Declaration of Helsinki, and the study was approved by the Ethics Committee of the Institute of Cognitive Neurology (INECO, now a host institution of the Institute of Cognitive and Translational Neuroscience).

**Table 1:** **Overall cognitive profile**.

	**CG**	**Controls (*n* = 6)**	***p*****-value**	***t*****-value**	**z_cc_**
Age	44	58.16 (6.73)	0.11	−1.94	−2.10
Education	18	15.83 (2.56)	0.47	0.78	0.84
IFS	25	24.83 (2.02)	0.94	0.07	0.08
ACE-R total	96	98.5 (1.97)	0.29	−1.17	−1.26
ACE-R subscales					
*Orientation*	10	9.83 (0.41)	0.73	0.37	0.41
*Attention*	8	8 (0)	–	–	–
*Memory*	23	24.33 (2.25)	0.61	−0.54	−0.59
*Verbal fluency*	13	13.67 (0.51)	0.28	−1.19	−1.29
*Language*	26	25.84 (0.41)	0.72	0.38	0.4
*Visual skills*	16	16 (0)	–	–	–

*Z_cc_, Z-scores; IFS, INECO Frontal Screening battery; ACE-R, Addenbrooke's Cognitive Examination Revised. Standard deviations are indicated between parentheses*.

### Neuroimaging: lesion localization

**Figure 1:**
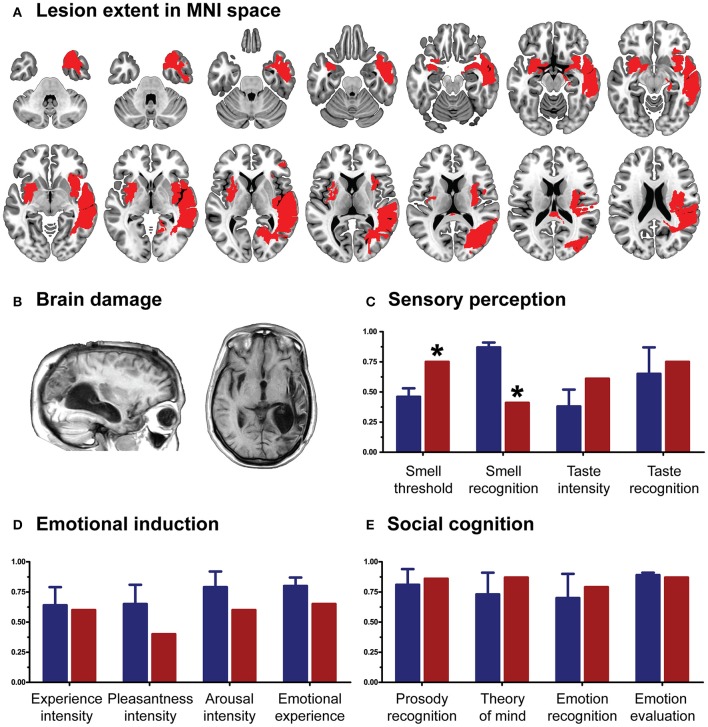
**(A)** Lesion extent in MNI space. Multislice overlap of lesions within a normalized brain from the MNI brain atlas. On the right hemisphere, these included the fronto-insulo-temporal cortices, spanning from the medial anterior temporal lobe (parahippocampal gyrus and amygdala) to the mid and superior temporal gyri; the supramarginal and angular gyri; the inferior parietal lobule; almost the complete insula; and a portion of the putamen and the inferior frontal operculum. On the left hemisphere, compromised regions included the left anterior insula and its underlying white matter, the putamen, and the dorso-lateral amygdala. **(B)** Brain damage. Original T1 sequence showing lateral and axial views of the patient's brain. **(C)** Sensory perception. Scores on tasks tapping smell and taste. **(D)** Emotional processing. Scores on a video-based emotional induction task inducing positive, negative, and neutral emotions. **(E)** Social cognition. Scores on tasks tapping emotional prosody recognition, theory of mind, facial emotion recognition, and emotional evaluation. Blue bars and lines represent controls' mean scores and standard deviations, respectively. Red bars represent the patient's scores. All scores are presented in percentage values. Asterisks (^*^) indicate statistical differences at *p* < 0.05.

As in previous works (García-Cordero et al., 2015, 2016; Melloni et al., 2016), the patient's lesion was manually traced in native space by an expert neurologist (BC) according to visible damage on T1 and T2 scans. Then, this traced mask was normalized to MNI space. Based on the overlap of this mask in an MNI brain template, the neurologist confirmed the extensive compromise of the right fronto-temporo-parietal cortices, left Sylvian and striatal regions, and bilateral portions of the insula and the amygdala (Figures 1A,B; see figure legend for anatomical details of the lesion).

### Instruments

The assessment protocol included multiple instruments tapping overall cognitive status (including orientation, attention, memory, verbal fluency, language, and visuospatial skills), sensory perception, emotional processing, anxiety levels, and social cognition.

#### Executive functions

Executive functions were assessed through the INECO Frontal Screening (IFS) battery (Torralva et al., 2009b), a sensitive tool for neurodegenerative disease assessment (Torralva et al., 2009a; Gleichgerrcht et al., 2011), in general, and medial frontal executive functions (Roca et al., 2011), in particular. Over a maximum total score of 30 points, a 25-point cut-off has shown a sensitivity of 96.2% and a specificity of 91.5% in detecting patients with dysexecutive syndrome (Torralva et al., 2009b). This test includes eight tasks: (1) motor programming: subjects perform the Luria series (“fist, edge, palm”), first by copying the administrator and then on their own; (2) conflicting instructions: subjects are required to tap the table once when the administrator taps it twice, or twice when the administrator taps it once; (3) motor inhibitory control: subjects are told to tap the table only once when the administrator taps it once, but to do nothing when the examiner taps it twice; (4) numerical working memory: subjects are asked to repeat a progressively longer string of digits in the reverse order; (5) verbal working memory: subjects are asked to list the months of the year backwards, starting with December; (6) spatial working memory: the examiner presents four cubes and points at them in a given sequence; the subject is asked to repeat the sequence in reverse order; (7) abstraction capacity: subjects are read proverbs and asked to explain their meaning; (8) verbal inhibitory control: this task, based on the Hayling test, measures the ability to inhibit an expected response; in the first part, subjects are read three sentences and asked to complete them correctly, as quickly as possible; in the second part, they are asked to complete another three sentences with a syntactically correct but semantically incongruous word. The maximum global score on the IFS is 30 points.

#### Overall cognitive state

The participants' overall cognitive state was evaluated with a version of the Addenbrooke's Cognitive Examination Revised, adapted for the Argentine population (Torralva et al., 2011). With a cut-off score of 85 points, this brief screening test has a sensitivity of 97.5% and a specificity of 88.5%. It comprises five subscales, tapping on attention/orientation (maximum score: 18), memory (maximum score: 26), fluency (maximum score: 14), language (maximum score: 26), and visuospatial skills (maximum score: 16). The maximum global score of the test is 100.

#### Taste

To evaluate taste intensity perception, we gave each participant five sapid stimuli at four increasing concentrations: sucrose (0.03, 0.1, 0.3, 1.0 M), sodium chloride (NaCl; 0.03, 0.1, 0.3, 1.0 M), citric acid (0.001, 0.003, 0.01, 0.032 M), and quinine hydrochloride (QHC1; 0.00003, 0.0001, 0.0003, 0.001 M). Each stimulus was dissolved in distilled water and presented at room temperature as part of an ascending concentration series, as in the Q-Tip test (Bartoshuk et al., 1985). With the subject's tongue extended and stabilized between the lips, each stimulus was applied to both sides of the anterior tongue using a sterile, cotton-tipped applicator. Participants used a number line (range = 0–10) to report the intensity of the stimulus before retracting their tongue. Subjects were told that the first stimulus of each concentration series, distilled water, rated zero on the taste intensity scale. The output score was intensity feeling (from 1 to 50), which, for the global taste intensity score, was later transformed into percentage values.

Then, to measure the subjects' ability to identify five basic tastants, we used the maximum concentrated stimuli from the previous task (0.3 M sucrose, 0.3 M NaCl, 0.01 M citric acid, and 0.0003 M QHC1 or distilled water) and applied them on their tongues following the same procedure described above. Each side of the tongue was tested two times for the five tastants. Participants indicated the perceived flavor by pointing to a labeled card in a five-option forced choice: salty, sweet, sour, bitter, or non-flavor. This test was conducted twice for each stimulus following procedures described elsewhere (Pritchard et al., 1999). The output score was correct responses from 1 to 10, which, for the global taste recognition score, was transformed into percentage values.

#### Smell

To establish odor sensitivity thresholds, we used eight solutions at increasing concentrations of phenyl ethyl alcohol in a staircase procedure based on the Sniffin' Sticks test (Hummel et al., 1997). Odor identification skills were assessed through the commercial test of olfactory function Brief Smell Identification Test (Doty et al., 1984), consisting of 12 stimuli with a forced-choice answer. Finally, threshold and identification means were used to create a global score variable representing overall smell performance.

Then, individual odor sensitivity was assessed by acquiring thresholds for phenyl ethyl alcohol with an ascending double-forced choice staircase procedure. We used an eight-step geometric series, starting from a 4% phenyl ethyl alcohol solution (dilution ratio 1:2 in deionized water). Each subject was presented for 3 s at a distance of 3 mm from each nostril with two bottles in a randomized order: one contained only the deionized water, and the other contained the odorant at a certain dilution. While blindfolded, the subjects were asked to identify the odor-containing bottle. The threshold was defined as the trial in which the participant correctly identified five consecutive stimuli (Hummel et al., 1997) and this number was later transformed to percentage of intensity of perceived smell.

Finally, odor identification abilities were further evaluated through the B-SIT (Sensonics Inc.). This test consisted of 12 stimuli, each presented for 3 s at 3 mm from each nostril. Each participant selected which odor was perceived from a forced-choice list with four options. The smell identification score was measured as the number of correct choices, ranging from 0 to 12, with higher scores indicating better identification. The 12 odors commonly used in commercially available tests were smoke, chocolate, onion, strawberry, gasoline, turpentine, banana, pineapple, cinnamon, soap, lemon, and rose. The number of correct responses was later transformed into an identification percentage.

#### Anxiety levels

Anxiety levels were assessed with the state subscale of the State Trait Anxiety Inventory (Spielberger et al., 1970), an introspective, self-report instrument widely used in research with adult populations. All 20 items of the subscale were included.

#### Emotional induction

Each participant watched nine affectively-laden clips (Feinstein, 2012) aimed to induce specific emotions classified as positive, negative, and neutral. Each clip was chosen according to previously published criteria, including brevity, self-containment, intensity, and specificity (Feinstein, 2012). Clips were presented in a pseudo-randomized order. Immediately after each clip, participants answered questions about peak intensity (“Experience intensity”), pleasantness (“Pleasantness intensity”), and arousal (“Arousal intensity”) of the experienced emotion. Finally, they completed the Positive and Negative Affect Schedule (Watson et al., 1988).

#### Emotional prosody recognition

Prosody recognition was assessed following previously reported protocols (Scott et al., 1997; Couto et al., 2013). Stimuli comprised six disyllabic concrete nouns with neutral meaning and spoken with six different intonations that intended to convey emotions of happiness, anger, fear, disgust, sadness, and surprise. Participants were presented with the stimuli binaurally and after each presentation, they were asked to identify the emotion by choosing one out of six options.

#### Theory of mind

Theory of mind was assessed through the Reading-the-Mind-in-the-Eyes (RTME) test (Baron-Cohen et al., 2001). Participants were presented with 36 gray-scale pictures showing cropped portraits focused on the faces' eyes. Four words denoting mental states are presented round each picture and participants must choose the word that best describes what the person is thinking or feeling. Only one response is correct for each trial. The maximum score is 36.

#### Facial emotion recognition

Facial emotion recognition was assessed via an e-morphing task (Hurtado et al., 2009; Couto et al., 2013). This computerized test features six basic emotions (happiness, surprise, sadness, fear, anger, and disgust) obtained from the Pictures of Affect Series (Ekman and Friesen, 1976). Each stimulus was morphed for each prototype emotion and for a neutral state (Young et al., 1997), and presented in 20 frames of 500 ms each. Participants indicated when they recognized each emotion by pressing a button.

#### Emotional evaluation

Emotional evaluation was assessed with the Test for the Awareness of Social Inference (TASIT) (McDonald et al., 2006), a sensitive social perception test to assesses the recognition of spontaneous emotional expressions (fearful, surprised, sad, angry, and disgusted) in short videos. This task introduces contextual cues of the social situation (e.g., speaker demeanor, including prosody, facial movement, and gestures) and additional processing demands (e.g., adequate speed of information processing, selective attention, and social reasoning) that are not taxed when viewing static displays. All scripts are neutral in content and do not lend themselves to a particular emotion. After viewing each scene, the test participant was instructed to choose the emotion expressed by the actor from a forced-choice list.

### Statistical analysis

Demographic and behavioral data of the patient and the controls were compared with Crawford's modified two-tailed *t*-test (Crawford and Howell, 1998; Crawford and Garthwaite, 2002, 2012; Crawford et al., 2009, 2011). This test is robust for non-normal distributions, presents low rates of type-I error, and has proved successful in previous single-case studies (Straube et al., 2010; Couto et al., 2013, 2014), even when the control sample comprises fewer than five subjects (Crawford, 1998). Alpha levels were set at *p* < 0.05.

## Results

CG's performance was similar to that of controls on almost every domain assessed. She showed no deficits in executive functions (*t* = 0.07, *p* = 0.94), overall cognitive state (*t* = −1.17, *p* = 0.29) or individual high-order subdomains (all *p*_s_ > 0.2)—for details, see Table 1. Also, her taste recognition (*t* = 0.4 *p* = 0.7) and intensity discrimination (*t* = 1.5, *p* = 0.2) skills were spared despite a reduced sense of smell (*t* = −9.28, *p* < 0.001)—for details, see Figure 1C and Table 2). Moreover, she was unimpaired in overall emotional experience processing (*t* = −1.07, *p* = 0.33), with preserved abilities to ascertain various aspects of both positive and negative emotions (all *p*_s_ > 0.33)—for details, see Figure 1D and Table 3. Finally, she evinced normal anxiety levels (*t* = 0.24, *p* = 0.82) and showed no disruptions of her social cognition skills, including prosody recognition (*t* = −0.26, *p* = 0.8), theory of mind (*t* = −1.47, *p* = 0.19), facial emotion recognition (*t* = −1.53, *p* = 0.17), and emotional evaluation (*t* = −1.86, *p* = 0.11)—for details, see Figure 1E and Table 4.

**Table 2:** **Sensory perception**.

	**CG**	**Controls (*n* = 6)**	***p*****-value**	***t*****-value**	**z_cc_**
Age	44	58.67 (6.65)	0.1	−2.04	−2.2
Education	18	15.83 (2.56)	0.47	0.78	0.84
Global taste intensity	61.25	38.58 (13.74)	0.2	1.5	1.64
Sweet recognition	2	1.2 (0.44)	0.17	1.63	1.78
Salty recognition	1	0.6 (0.89)	0.7	0.4	0.44
Acid recognition	2	2 (0)	–	–	–
Bitter recognition	1	1 (0.7)	1	0	0
Global taste recognition	75	65 (22.36)	0.7	0.4	0.44
Smell threshold	75	45.83 (6.97)	0.01	3.87	4.18
Smell recognition	41.67	87.5 (4.56)	>0.001	−9.28	−10.03

*Z_cc_, Z-scores. Taste was assessed with the Q-tip test. Smell was assessed with the Sniffin' Sticks test. Standard deviations are indicated between parentheses. Data missing for one subject on the Taste test*.

**Table 3:** **Anxiety levels and emotional induction**.

	**CG**	**Controls (*n* = 6)**	***p*****-value**	***t*****-value**	**z_cc_**
Age	44	50 (10.63)	0.62	−0.52	−0.56
Education	18	18.67 (1.21)	0.63	−0.5	−0.55
**ANXIETY**					
STAI-S	39	36 (11.42)	0.82	0.24	0.26
**ALL EMOTIONS**					
Experience intensity	43.25	40.91 (6.82)	0.46	0.31	0.34
Pleasantness intensity	3.5	2.91 (0.73)	0.49	0.73	0.79
Arousal intensity	4.75	4.22 (1.25)	0.72	0.38	0.41
Emotional experience	104.5	107.25 (2.38)	0.33	−1.07	−1.15
**POSITIVE EMOTIONS**					
Experience intensity	50.5	53 (4.13)	0.59	−0.56	−0.6
Pleasantness intensity	4	5.67 (2.16)	0.5	−0.71	−0.77
Arousal intensity	4.5	4.58 (2.08)	0.97	−0.03	−0.03
Emotional experience	109	124.41 (14.14)	0.35	−1	−1.09
**NEGATIVE EMOTIONS**					
Experience intensity	34.75	29.7 (11.5)	0.7	0.4	0.43
Pleasantness intensity	1.5	1.12 (0.94)	0.72	0.37	0.39
Arousal intensity	4.75	5.54 (2)	0.72	−0.36	−0.39
Emotional experience	93.5	96.31 (6.55)	0.7	−0.4	−0.42
**NEUTRAL EMOTIONS**					
Experience intensity	53	51.25 (7.27)	0.83	0.22	0.24
Pleasantness intensity	7	3.75 (1.17)	0.05	2.56	2.77
Arousal intensity	5	1.25 (1.29)	0.04	2.68	2.89
Emotional experience	122	111.25 (9.73)	0.35	1.02	1.1

*Z_cc_, Z-scores. STAI-S, state version of the State Trait Anxiety Inventory. Emotional induction was assessed with the video-based paradigm reported in Feinstein (2012). Standard deviations are indicated between parentheses*.

**Table 4:** **Social cognition**.

	**CG**	**Controls (*n* = 8)**	***p*****-value**	***t*****-value**	**z_cc_**
Age	44	51.12 (7.16)	0.37	−0.93	−0.99
Education	18	13.87 (3.97)	0.36	0.97	1.03
Prosody recognition	0.6	0.64 (0.15)	0.8	−0.26	−0.28
Theory of mind (RTME)	14	23.5 (5.95)	0.19	−1.47	−1.59
Facial emotion recognition (E-morphing)	0.58	0.79 (0.12)	0.17	−1.53	−1.62
Emotional evaluation (TASIT)	13	16.14 (1.57)	0.11	−1.86	−1.99

*Z_cc_, Z-scores. Prosody recognition was assessed following the protocol reported in Couto et al. (2013) and Scott et al. (1997). Theory of mind was assessed through the Reading-the-Mind-in-the-Eyes (RTME) test. Facial emotion recognition was assessed via an e-morphing task. Emotional evaluation was assessed with the Test for the Awareness of Social Inference (TASIT). Data missing for two subjects on the prosody recognition and RTME tests, and for one subject on the TASIT. Standard deviations are indicated between parentheses*.

## Discussion

One would expect CG's extensive pattern of neural damage to have severely compromised her functionality. Indeed, cross-sectional and longitudinal evidence shows that lesion site information predicts the severity of specific cognitive deficits after stroke (Hope et al., 2013). In this sense, the patient exhibited various patterns of damage known to systematically disrupt various domains. For example, putative associations have been established between frontostriatal lesions and motor disability (Zgaljardic et al., 2003), insulo-parietal compromise and somatosensory deficiencies (Meyer et al., 2016), bilateral fronto-insular damage and social cognition deficits (Ibáñez et al., 2010, 2016a; Couto et al., 2013; Baez et al., 2014, 2016b; Melloni et al., 2016), bilateral frontal atrophy and executive dysfunctions (Rabinovici et al., 2015), left perisylvian lesions and language impairment (Ullman, 2008), and right-sided frontal disturbances and altered pragmatic skills (Kaplan et al., 1990; Stemmer, 2008). Moreover, the regions affected should typically disrupt multiple functional networks, such as the salience network (Seeley et al., 2007), the social context network (Ibáñez and Manes, 2012; Baez et al., 2016a), and the fronto-parietal attention network (Ptak, 2012).

These mappings, though subject to some inter-individual variability at a relatively fine scale, are remarkably consistent and systematic across subjects. However, formal assessments revealed almost none of the expected deficits. CG's performance was similar to that of matched controls on tasks tapping executive functions and overall cognitive state (e.g., attention, memory, language). Also, her taste was preserved despite a reduced sense of smell, and she was unimpaired in emotional processing and social cognition skills.

Additional data gleaned during neurological examinations further confirm CG's preserved abilities. She has intact motor skills: she routinely types on her computer, walks at least 1 h each morning, and goes swimming every week. Likewise, except for hyposensitivity on her right hand and a reduced sense of smell, her sensory abilities (e.g., auditory perception, taste, temperature processing, skin sensitivity, proprioception) are completely spared. For example, she could perfectly identify sounds within and outside the meeting room, and on one occasion she reasonably complained that the coffee was too hot and sweet; also no other tactile or somatosensory alterations were mentioned in her clinical history or in the several interviews conducted in the course of the study. Moreover, her emotional repertoire seems uncompromised: during the interviews she exhibited normal basic and social emotions, and she provided clear examples of recent scenarios in which she predictably experienced joy, fear, frustration, exultation, and other feelings. She is also capable of inferring other people's beliefs and emotional states, as seen, for instance, in her detection of anxiousness when two of the present authors were readying themselves for a book presentation. Additionally, her interpersonal behavior is consistently adequate during interactions with the institution's staff, her caregivers, and her children, except for a tendency to be overly open regarding her personal problems (she is unreserved in discussing her lesions, her feelings, and the social difficulties she has encountered, even with people she meets for the first time). However, no signs of hypomania were observed in her clinical history or in the several interviews she granted us in the course of the study.

Linguistically and pragmatically, she gives no signs of impairment: her articulatory, phonological, lexical, and grammatical abilities are fully preserved, as are her prosodic and turn-taking capacities (let alone her knack for bantering, construing metaphors, and introducing ironic remarks). Even more striking are her mnemonic skills: her procedural and semantic knowledge is fully preserved, as shown in her smooth execution of various action routines (e.g., handling her cell phone, tying her shoe laces) and her intact naming and classification skills (e.g., she could flawlessly denominate all the objects she had thematically organized in different sections of her purse); furthermore, her declarative memory is extremely detailed for events which happened weeks, months, and even years ago. She could describe scenes from her childhood and adolescence, she meticulously narrated episodes occurring immediately before and after her strokes, she remembered the names, specialties, and suggestions of all her doctors, and she could recount details of dozens of books she had read throughout her life. Finally, her executive functions (including attention, working memory, and prospective thought) are notably acute. During our lengthy interviews she remained focused, efficiently resumed topics interrupted by the flow of dialog, and sensibly discussed her plans and concerns for the future. Nevertheless, a mild inhibitory deficit may underlie the above-mentioned tendency to discuss private parts of her life without reservations, even with people she has never met before.

Still, the most compelling demonstrations of her functional integrity emerged outside formal assessment settings. In the quest of third-party impressions, two of the present authors (AMG and AI) visited the patient's home to interview her mother and a long-time friend. Both caregivers confirmed our observations and were unable to identify any significant impairment. The most eloquent proof, however, was CG's resourcefulness as a host. Her preparation of various beverages and snacks, handling of trays and cups, and coordination of the conversation attested to her capacity to efficiently fulfill multiple concurrent tasks even under the shifting demands of a complex, context-rich setting.

Therefore, this case resists straightforward classifications. While CG presents some deficits (e.g., right-hand hyposensitivity, smell deficiencies), the extent and distribution of her lesions and the nature of their etiologies would lead one to expect much more widespread deficits. Seen under this light, the case constitutes an anomaly, since it defies most expectations and theories about neurocognition.

CG adds to an interesting corpus of case studies challenging mainstream accounts of the relationship between brain anatomy and function. For example, Feuillet et al. (2007) reported on a civil servant with hydrocephalus, who had an IQ of 75, fully preserved everyday functionality, and normal medical and neurological development despite progressive loss of nearly three fourths of his brain. His maintenance of low- and high-level cognitive functions, including consciousness, has puzzled scientists for almost a decade. Also relevant are the cases of a 58-year-old patient with subtotal cerebellar agenesis and a completely normal neurological profile (Sener and Jinkins, 1993) and that of a 24-year-old woman who was born without a cerebellum and had only mild symptoms in functions associated with such a structure (Yu et al., 2015): despite slight delays in her development of oral and locomotive skills and moderate signs of dysarthria and dysmetria, this patient had intact orientation and motor independence, while giving no signs of sensory or linguistic dysfunction. Both individuals deviate from the widespread pattern of impairments typically observed in patients with primary cerebellar agenesis—for a review, see Yu et al. (2015).

Like the studies cited above, CG's case does not find a straightforward explanation in mainstream views of neurocognition. First, her lesion was acquired late in life, which precludes the operation of early compensatory mechanisms present in cases of agenesis or tissue removal in infancy or childhood. Alternatively, CG might have recruited completely atypical circuits since birth to process each of her preserved functions, so that her lesions were not actually compromising putative hubs. Yet, this is also unlikely, given that her pattern of damage extended across multiple cortical and subcortical areas bilaterally, which leaves little chances for possible uncompromised mechanisms subserving such functionally varied domains.

Admittedly, we cannot fully rule out overall compensatory brain changes, since much of the evidence we reported was obtained roughly 1 year after CG's second stroke. However, favorable post-stroke rewiring could hardly account for the case in its entirety. Indeed, plastic mechanisms in several regions (particularly, the prefrontal and hippocampal cortices) prove vulnerable to aging, due to changes in neuronal morphology and connectivity, among other factors (Burke and Barnes, 2006). It would thus seem unlikely for multiple functions to have become near-optimally subserved by compensatory or alternative mechanisms only a few months after adult-onset strokes.

Second, we have entertained the hypothesis that this may be an extreme case of cognitive reserve, the brain's capacity for functional resilience following damage or throughout healthy aging (Stern, 2012). Indeed, this conjecture was nurtured by the fact that CG features many possible neuroprotective factors. She has never smoked or drunk alcohol and always kept a balanced diet. She has high educational achievements and is fluent in two languages. Since childhood, she constantly engaged in logical games and mindfulness-like exercises, practiced several sports, developed artistic skills, engaged in extracurricular scientific studies, and joined different cliques in each of these areas. Nevertheless, the case would prove anomalous even if seen under this light. Indeed, none of her potentially neuroprotective experiences was unusually intense (she was an average student, her artistic prowess is within the norm of an amateur, and her use of a second language was confined to very specific scenarios and only during a few years of her life). Likewise, neither does the absence of risk factors seem enough to explain the full preservation of her cognitive, emotional, and behavioral skills (as opposed to just one specific subdomain) upon acquiring severe lesions in putative neural substrates. However, the *combination* of these features might have contributed to her resilience via a cumulative effect—though this remains speculative.

Third, it may be that the cardiac arrest and/or the second stroke suffered by CG paradoxically contributed to *resolving* deficits triggered by her first episode. This conjecture follows from both animal and human research. For example, serial lesions of the prefrontal association cortex in rhesus monkeys can induce milder behavioral impairments than one-stage lesions of the same or less severity (Rosen et al., 1971). Similarly, highly specific cognitive abnormalities (e.g., foreign accent syndrome) triggered by a left frontoparietal infarct have been observed to disappear following a second stroke compromising contralateral regions (Cohen et al., 2009). Unfortunately, this possibility cannot be directly assessed since there is no behavioral data on CG's cognitive skills after her first stroke. However, even if this were a feasible explanation of the present case, it would still prove hard to accommodate within most frameworks in cognitive neuroscience.

Finally, it could be that many or all of the above factors were implicated in CG's atypical anatomo-clinical profile. Should that be so, our main contention would remain, as no extant theory offers an explicit integrated account of late-onset multidimensional plasticity, experience-based cognitive reserve, and paradoxical recovery triggered by a second lesion. If anything, this patient exposes the limitations of our current understanding of brain organization and resilience.

## Conclusion

In sum, CG highlights the importance of considering individual cases to challenge our assumptions about neurocognition. Although our work is limited because of the absence of functional imaging data, her widespread preservation of cognitive functions despite extensive acquired damage merits scholarly attention. Beyond our current theories of brain plasticity, compensatory mechanisms, or cognitive reserve, there seem to be hitherto unknown forms of functional resilience. Reporting on unusual patients and disseminating the puzzling findings they offer contributes to fostering new avenues of research and thus inspire both theoretical and translational developments in the field.

## Author contributions

AI and AG designed the study. AI, AG, LS, EM, BC carried out the experiments. AI, AG, LS, EM, BC analyzed the experiments. AG, AI, LS wrote the paper. AI, AG, BC contributed to the clinic aspects of the paper. AG and IA conceived the study and wrote the final paper, together with the other authors. All authors have approved the manuscript.

### Conflict of interest statement

The authors declare that the research was conducted in the absence of any commercial or financial relationships that could be construed as a potential conflict of interest.
